# The Role of Chicken Prolactin, Growth Hormone and Their Receptors in the Immune System

**DOI:** 10.3389/fmicb.2022.900041

**Published:** 2022-07-14

**Authors:** Guodong Mo, Bowen Hu, Ping Wei, Qingbin Luo, Xiquan Zhang

**Affiliations:** ^1^Guangdong Provincial Key Laboratory of Agro-Animal Genomics and Molecular Breeding, College of Animal Science, South China Agricultural University, Guangzhou, China; ^2^Key Lab of Chicken Genetics, Breeding and Reproduction, Ministry of Agriculture, Guangzhou, China; ^3^State Key Laboratory for Conservation and Utilization of Subtropical Agro-Bioresources, South China Agricultural University, Guangzhou, China; ^4^Institute for Poultry Science and Health, Guangxi University, Nanning, China

**Keywords:** chicken, growth hormone, immune response, prolactin, receptor

## Abstract

Prolactin (PRL) and growth hormone (GH) exhibit important roles in the immune system maintenance. In poultry, PRL mainly plays its roles in nesting, hatching, and reproduction, while GH is primarily responding to body weight, fat formation and feed conversion. In this review, we attempt to provide a critical overview of the relationship between PRL and GH, PRLR and GHR, and the immune response of poultry. We also propose a hypothesis that PRL, GH and their receptors might be used by viruses as viral receptors. This may provide new insights into the pathogenesis of viral infection and host immune response.

## Introduction

Hormones like prolactin (PRL) and growth hormone (GH) are highly effective biological active substances secreted by secretory gland organs or endocrine cells, which are closely related to the regulation of the nervous system, internal environment stability and various body functional activities (Hiller-Sturmhöfel and Bartke, 1998; Bahadoran et al., 2019). PRL and GH play an essential role in regulating metabolism, growth, development, and reproduction, as well as directly or indirectly affect individual various systems, including digestive, reproductive, endocrine, and immune systems (Bahadoran et al., 2019). In poultry, PRL and GH are related to growth, development, and reproduction (Nie et al., 2005; Qin et al., 2013; Wilkanowska et al., 2014). However, some studies have shown that after chicken infected with some pathogens, such as Eimeria tenella (*E. tenella*) (Chadwick et al., 1985; Davison et al., 1985), Histomonas meleagridis (Chadwick et al., 1980), infectious bronchitis virus (IBV) (Nii et al., 2015), Newcastle disease virus (NDV) (Rehman et al., 2020), infectious bursal disease virus (IBDV) (He et al., 2018; Yu et al., 2019), Marek's disease virus (MDV) (Liu et al., 2001) and avian leukosis virus (ALV) (Carter and Smith, 1983, 1984; Dai et al., 2019; Mo et al., 2021, 2022), it is found that the hormone levels are changed, and they are involved in regulating the body immune response (Allen et al., 1997; Breuel et al., 2004; Hartwell et al., 2014; Yu et al., 2020; Hu et al., 2021). These evidences suggest that hormones are involved in the immune responses under specific conditions, which present an often-overlooked challenge to the conventional idea that hormone merely as endocrine hormone.

Avian leukosis (AL) has caused great harm to the poultry industry. So far, there are no effective vaccines and drugs, and the harm of AL can only be eradicated through population purification measures. The time of AL purification is mainly carried out at the initial stage of egg production and the peak egg production period of the flock. When tested during these two periods, the positive rate for avian leukosis was higher than for other growth cycles (Zhang et al., 2010). During these two periods, some hormones in chickens will undergo major changes. Studies have shown that plasma levels of hormones are closely related to viremia (Dai et al., 2019). We performed RNA-seq of spleens from fast- and slow- feathering chickens infected with ALV-J (PRJNA552417), and found that the *PRL* genes were enriched in the cytokine-cytokine receptor interaction, and systemic lupus erythematous signaling pathways. In our previous study, after virus infection, the levels of PRL and GH in plasma have changed and the increase PRL levels could reduce the expression of virus and pro-inflammatory factors in immune organs (Mo et al., 2021, 2022).

Hyperprolactinemia is associated with autoimmune suppressive diseases in humans and affects their pathogenesis, such as systemic lupus erythematosus and rheumatoid arthritis (Fojtíková et al., 2010; Borba et al., 2018). Although the mechanism of their interaction is not fully understood, previous researches suggest that PRL can influence and regulate the body's immune response (Borba et al., 2018; Recalde et al., 2018). PRL is a polypeptide hormone expressed in various immune cells and tissues, which has regulatory function on reproductive endocrinology, growth, metabolism, behavior, and immunity (Hiller-Sturmhöfel and Bartke, 1998; Bahadoran et al., 2019). It acts as a cytokine to modulate immune responses through paracrine and autocrine mechanisms (Gala, 1991; Borba et al., 2018; Recalde et al., 2018). After binding to the PRL receptor (PRLR), PRL activates multiple pathways, including Janus kinase/signal transducers and activators of transcription (JAK/STAT), mitogen-activated protein kinase (MAPK) and phosphoinositide-3-kinase/AKT (PI3K/AKT) pathways (Fojtíková et al., 2010). PRL and GH are similar in structure and function, which belong to the same hormone family and originate from a common ancestor (Boutin et al., 1988). PRL and GH along with their receptors share structural similarities with cytokines and their receptor superfamilies, suggesting that they are evolutionarily and functionally related to the immune system (Bazan, 1989; Goffin and Kelly, 1997; Yada, 2007). GH also activates JAK/STAT, MAPK and PI3K/AKT signaling after binding to the GH receptor (GHR) (Hu et al., 2021). However, the effects of GH on the immune system remain controversial. Some studies have linked it to the autoimmune suppression (Murphy et al., 1992; Gonzalo et al., 1996; Villares et al., 2013). PRL and GH deficiency can cause impaired host immune function (Gala, 1991). Based on the above study, we speculate that PRL and GH play an essential similar role in poultry immune response after virus infection.

In this review, we summarized the roles of PRL and GH in the poultry immune response, respectively. We then discussed the roles of PRL and GH receptors in immunity to determine whether they are indispensable factors in immune regulation. In addition, we attempt to provide a critical overview on the roles of the two hormones in the immune response in poultry, exploring their contribution to the development of diseases. Specially, we also emphasize the challenges between the two hormones and the immune system.

## Prolactin and the Immune System

PRL is a 23 kDa polypeptide hormone secreted by anterior pituitary cells, which has high homology in different poultry varieties (Kansaku et al., 2008; Wang et al., 2009). PRL also exists in thymus, spleen, lymphocytes, and epithelial cells (Bolefeysot et al., 1998; Wilkanowska et al., 2014). The chicken PRL is mainly related to nesting, hatching, egg production, broodiness (Jiang et al., 2005; Cui et al., 2006; Rashidi et al., 2012; Li et al., 2013; Wilkanowska et al., 2014).

The levels of PRL in plasma is different in various growth periods of chicken (Jiang et al., 2005; Wilkanowska et al., 2014). Even before and after ovulation, there are significant changes (^*^P < 0.05) in the PRL concentrations (Scanes et al., 1977). Meanwhile, PRL levels alters in different chicken breeds (Mo et al., 2022). When chickens are infected with pathogens, such as Histomonas meleagridis (Chadwick et al., 1980), *E. tenella* (Chadwick et al., 1985), and ALV-J (Mo et al., 2021), the plasma PRL levels have changed compared with the uninfected chickens. These results indicate that PRL levels is not only affected by individual own growth cycle but also controlled by external factors.

PRL is a cytokine that stimulates cellular and humoral immunity at the same time. At the 14th days post-infection (dpi) infected with Trypanosoma cruzi (*T. cruzi*), PRL treatment increases the percentage of natural killer (NK) cells and B lymphocytes in the rat spleen compared to the infected without PRL treatment and the untreated group (Filipin et al., 2019). Studies have revealed that PRL act as an immune cells regulator in parasite infection, strengthen the host immune system, and reduce the pathological effects of pathogens (Filipin et al., 2011, 2019; Río-Araiza et al., 2018a,b). PRL promotes lymphocyte mitogenesis isolated from thymus and spleen of White Leghorn chickens in a dose-dependent manner (SkwarłoSońta, 1990). Different doses of ovine PRL were used to treat chicken bursa of fabricius cells, and all doses of PRL increase the mitotic activity of cells, and the lowest dosage of PRL is most effective (Bhat et al., 1983). Moreover, PRL modified the difference in the chemotactic factor and the leukocyte migration of fetal membranes in a tissue-specific manner (Núñez-Sánchez et al., 2021). Our previous results showed that pre-incubating DF-1 cells with PRL before ALV-J infection elicits the best antiviral effects regardless of dose *in vitro* (Mo et al., 2021). Moreover, PRL can reduce the expression of pro-inflammatory cytokine-encoding *TNT*α, *IL-1*β, and *IL-6* genes, and increase the expression of the interferon-stimulated genes of oligoadenylate synthetase-like (*OSAL*) and vasoactive intestinal peptide (*VIP*) in the spleens of ALV-J-infected chicks (Mo et al., 2021). Interestingly, hormone deficiency can alter the host immune system and reduce specific antibodies against pathogens (Quintanar-Stephano et al., 2015; Hernández-Cervantes et al., 2018; Río-Araiza et al., 2018a,b). The differences of PRL levels among breeds may also be responsible for the differences in virus susceptibility among breeds (Mo et al., 2022). PRL plays an important role in regulating and maintaining the host immune response, and is an indispensable part of the host immune system.

During the lactation, PRL can increase the number of immune cells in mammary secretions and enhance the chemotaxis effect on T cells, B cells, monocytes, and macrophages (Dill and Walker, 2017). In addition, PRL can reduce the activation threshold of B cell receptors and induce *CD40* expression in B cells (Correale et al., 2014). During the first hour of bacterial infection, PRL increases the expression of *TLRs* and *MyD88*, while the expression of *IL-1*β is continuously increased (Peña et al., 2016). Pituitary cells can directly recognize the fungal cell wall glucans to promote the expression of *TLR4* and *CD14* (Breuel et al., 2004). During *T. cruzi* infection, PRL increase the NK cells levels in treated infected animals compared to the untreated group (Filipin et al., 2019). Furthermore, PRL could reduce lipopolysaccharide (LPS)-induced inflammatory cytokines (TNF-α, IL-1β, and IL-6) via inhibiting NFκB phosphorylation and TLR4 expression (Olmos-Ortiz et al., 2019). However, the inhibitory effect of PRL is selective (Flores-Espinosa et al., 2020). PRL also stimulates the internalization of staphylococcus aureus on bovine mammary epithelial cells, and up-regulates the mRNA expression of *TNF-*α, *IL-1*β, and inducible nitric oxide synthase (Gutiérrez-Barroso et al., 2008). PRL constrains tumor-promoting liver inflammation by inhibiting MAP3K-dependent activation of c-Myc at the level of the “trafasome” (comprised of IRAK1, TRAF6, and MAP3K proteins) (Hartwell et al., 2014). However, the macrophages exposed with PRL secrete more inflammatory factors and reactive oxygen species, thereby aggravates the inflammatory response (Majumder et al., 2002; Sodhi and Tripathi, 2008; Tang et al., 2016). These results suggest that PRL not only affects the differentiation, regulation, and responsiveness of immune cells, but also the secretion of different immune messengers, such as chemokines, interleukin, and interferon, and increases layers of complexity to the interactive molecular and cellular events that occur in inflammatory and virus infectious diseases (Recalde et al., 2018).

PRL gene transcription is activated by the cyclic adenosine monophosphate (cAMP)-induced factor(s) and pituitary-specific transcription factor 1 (Pit-1) (Ohkubo et al., 2000). PRL secretion in poultry is under stimulatory control exerted by the hypothalamus (AlKahtane et al., 2003; Wilkanowska et al., 2014). Therefore, PRL secretion is predominantly regulated by the VIP, dopamine (DA), and serotonine (5-HT) (Macnamee et al., 1986; Kagya-Agye et al., 2012). VIP is a pleiotropic neuropeptide released by neurons and immune cells, which is widely distributed and expressed in all tissues and organs, and plays an important role in inflammation and autoimmune suppression diseases (Delgado and Ganea, 2011; Ganea et al., 2014). The viral infection affects the expression of *VIP* in immune organs. After chicks were infected with ALV-J, the *VIP* mRNA expression in the spleen was correlated with the levels of PRL in plasma (Mo et al., 2021).

Many cytokines are produced in the brain, hypothalamus, or pituitary gland, such as interferon (IFN)-α, IFN-γ, IL-1, IL-2, IL-6, IL-18 and TNF-α (Breder et al., 1988; Petrovsky, 2001; Silverman et al., 2005; Borghetti et al., 2009). These factors can stimulate or inhibit hormone secretion in the central nervous system (Rothwell and Hopkins, 1995; Steinmann, 2004; Borghetti et al., 2009). Furthermore, chemokines and their receptors also play regulatory roles in the neuroendocrine system (Callewaere et al., 2007). IL-1, IL-2, and IL-6 can stimulate PRL secretion, while endothelin-3 and IFN-γ play an inhibitory influence (Chikanza, 1999; Borba and Shoenfeld, 2019). The fungal cell wall glucans also promote the secretion of PRL (Breuel et al., 2004). LPS might promote the secretion of PRL by anterior pituitary cells through the mediation of IL-6 (Tomaszewska-Zaremba et al., 2018). However, cytokines IL-1β, IL-2, and IL-4 reduce *PRL* expression in T lymphocytes (Gerlo et al., 2005). ALV-J causes chicken monocyte cells death and accompanies with increased IL-1β and IL-18 expression (Dai et al., 2017). Moreover, the *PRL, IL-1*β, and *IL-6* expression is also higher than that of uninfected individuals (Mo et al., 2021). Apparently, cytokines act as a two-way communication between the immune and endocrine systems. There might be a possible that the elevated expression of pro-inflammatory cytokines induced by virus infection may affect the PRL expression *in vitro*. The expression of proinflammatory factors (mainly IL-1, IL-6, and TNF-α) is up-regulated during the acute phase reaction (APR) in infection. These pro-inflammatory cytokines stimulate the neuroendocrine system, thereby enhance innate immunity, induce metabolic changes, and control inflammation to restore body balance (Berczi et al., 1998). In fact, cytokines produced during infection indeed play a regulatory role in coordinating the endocrine and immune systems.

PRL is not only a pituitary hormone that plays a major role in reproduction, but also acts as a cytokine in the immune response. PRL can influence the local environment of immune organs and contribute to the maturation and function of immune cells. The presence of *PRL* significantly improves the proliferation of immune cells (Fojtíková et al., 2010). However, the effects of PRL on the immune system are contradictory. It inhibits lymphocyte proliferation at high concentrations and enhances proliferation at low concentrations (Matera et al., 1992; Nela et al., 2014; Suarez et al., 2015). In CD4+ T cells, low-dose of PRL induces *T-bet* expression, but high-dose of PRL exhibits an opposite effect (Tomio et al., 2008). It means that different doses of PRL will have different effects on downstream responses (Tomio et al., 2008; Zhang et al., 2019). Therefore, it is possible that PRL regulates host immunity over a very narrow range of PRL concentrations. The correlation between PRL and autoimmune diseases such as systemic lupus erythematosus, rheumatoid arthritis, systemic sclerosis, and multiple sclerosis, has been confirmed, in which the excessive of PRL expression aggravates the symptoms of autoimmune diseases (Vera-Lastra and Jara, 2002; Borba et al., 2018; Borba and Shoenfeld, 2019). Thus, this situation may also exist in poultry. The high expression of PRL may enhance the immune response of birds, but the continuous high expression may lead to the occurrence of autoimmune diseases in poultry.

**Figure 1:**
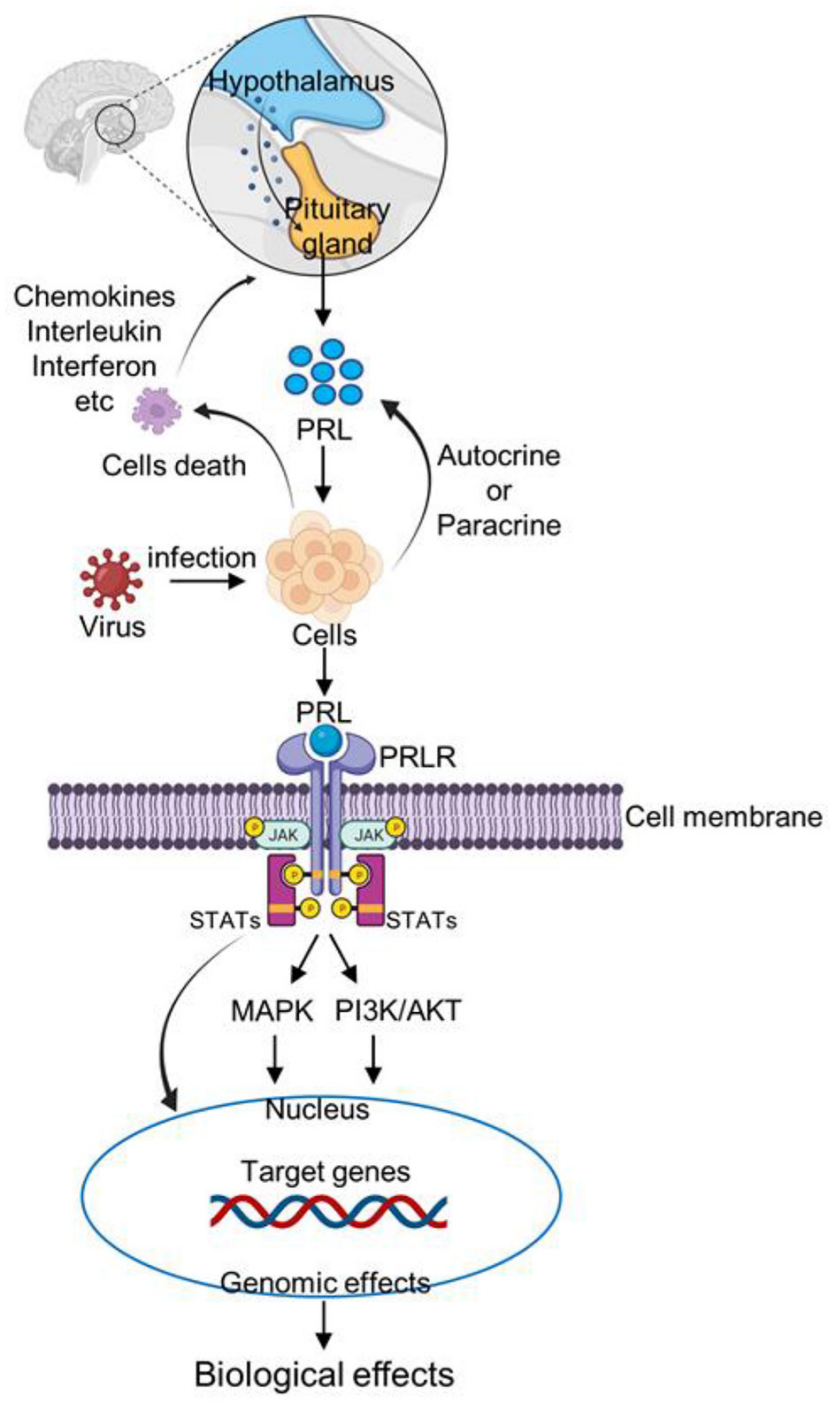
Schematic representation of PRL signaling pathway after virus infection. After infecting host cells, the virus causes immune cells death and increase the expression of inflammatory factors, such as chemokines, interleukin, and interferon. Inflammatory factors stimulate the pituitary gland to increase PRL secretion. In addition, cells can increase PRL secretion by autocrine or paracrine pathways. Then PRL binds to the PRLR receptor on the cell membrane, and activates JAK/STAT, MAPK, and PI3K signaling pathways, thus regulating the individual immune response.

PRL itself cannot initiate the immune response, and mainly maintains the balance of immune response in the body (Fojtíková et al., 2010). PRL can triggers different reaction pathways (Gala, 1991; Bolefeysot et al., 1998). Activation of these pathways results in endpoints such as differentiation, proliferation, survival, secretion, and immune (Bolefeysot et al., 1998; Kansaku et al., 2008). This supports the hypothesis that when pathogens invade and attack host cells, cell damage or death leads to increase the expression of pro-inflammatory cytokines such as interleukins, chemokines, interferons, and tumor necrosis factors, and then the pro-inflammatory cytokines increase or inhibit PRL expression/secretion by regulating hypothalamus or immune cells. The combination of PRL and PRLR activates JAK/STAT, MAPK, and PI3K signaling pathways, thus regulating the individual immune response (Figure 1). However, there are still many mechanisms in this process that remains unknown, such as the mechanism by which pro-inflammatory factors promote or inhibit the secretion of PRL? When facing different pathogens, which signaling pathway is activated by PRL?

## Growth Hormone and the Immune System

Growth hormone (GH) is a 22 kDa single chain polypeptide hormone synthesized and secreted by eosinophils in the anterior pituitary. The GH gene sequences in different poultry breeds have high homology, but low homology compared with mammals (Lamb et al., 1988; Tanaka et al., 1992). Except for pituitary gland, GH also expresses in various tissues and cells, such as spleen, thymus, ovary, kidney, liver, and lymphocytes (Gala, 1991; Liu et al., 2001). These results suggest that in addition to the endocrine mode of action, there is an autocrine or paracrine mode in tissues and cells. GH accelerates muscle and bone growth, protein synthesis and fat decomposition in animals. Moreover, GH regulates gender differentiation, sexual maturity, pregnancy, lactation, and reproduction (Stephen et al., 2001; Rotwein and Chia, 2010).

The secretion of GH is directly regulated by the hormones of hypothalamus, such as growth hormone releasing hormone (GHRH) and somatostatin (SRIF). Ghrelin, a GH-releasing peptide, is a powerful endogenous GH-secretin (Peino et al., 2000; Seoane et al., 2000). Ghrelin can induce GH secretion by activating the growth hormone secretagogue receptor in the hypothalamus and pituitary gland (Reichenbach et al., 2012). IL-1 has a direct effect on the hypothalamic-pituitary-adrenal (HPA) axis at both the hypothalamic and pituitary level, leading to increase in plasma concentrations of GH, luteinizing hormone (LH), and follicle-stimulating hormone (FSH) (Sapolsky et al., 1987; Beach et al., 1989). TNF-α can induce GH secretion in APR (Berczi et al., 1998; Borghetti et al., 2009). GH can promote the IL-6 expression in the thymus, while IL-6 can stimulate hypothalamus and induce the pituitary to secrete GH (Tsigos et al., 1997). As IL-6 receptors are distributed in the brain, pituitary gland, and adrenal gland (Hopkins, 2007), GH may respond rapidly to innate immune responses to pathogens by increasing levels of related cytokines. Cytokines act as a molecular signal transduction between GH secretion and the immune system. Other factors, such as breed, age, and other conditions (temperature, light, and nutrition level) also affect the GH secretion (Harvey et al., 1978; Castaño et al., 2005).

Like the expression of PRL, viral infection also affects the expression of GH in plasma. Five-week-old piglets immunized with porcine reproductive and respiratory syndrome virus (PRRSV) exhibit the increased levels of GH, pro-inflammatory and pro-immune cytokines in the plasma compared with the control group (Borghetti et al., 2011). The plasma levels of GH in 4-week-old piglets infected with highly pathogenic PRRSV is much higher than that of blank control group and conventional strain group at 7–21 dpi (Saleri et al., 2019). After infection with human immunodeficiency virus (HIV), the spontaneous GH secretion of patient and the GH response to the stimulation are attenuated (Rochira and Guaraldi, 2017). In antiretroviral therapy (cART), the combined use of GH can reduce the body's immune activation, improve CD4+ T lymphocyte count and HIV-1-specific T cell response (Herasimtschuk et al., 2014). The mice with generalized ablation of GHRH gene (Ghrh -/-) are highly susceptible to Streptococcus pneumoniae, but have normal thymus and T-cell development (Bodart et al., 2018). After ALV-J infection, the plasma of GH levels is higher than that of uninfected chickens (Mo et al., 2022). The interaction between GH and the immune system has been demonstrated in a variety of domestic animals (Borghetti et al., 2009). These results indicate that GH also plays an important role in the immune system of chickens. GH can regulate the immune response of the body. Treatment of autoimmune thyroiditis with recombinant chicken GH increases the proportion of CD4+ and CD8+ in thymocytes (Marsh et al., 1992). GH affects the growth and development of thymus and the maturation of lymphocytes in thymus, but has no effect on bursa of fabricius (Johnson et al., 1993). Continuous injection of GH into chicks for a week, the blastogenic response of lymphocytes to concanavalin-A or LPS mitotic stimulation is significantly increased (Haddad and Mashaly, 1992). Recombinant bovine GH (rbGH) can be used as an immunomodulator against *E. tenella* infection in chickens, but has no effect on *E. cervulina* or *E. maxima* (Allen et al., 1997). However, the rbGH regulates the weight growth of chicks depending on the dosage (Allen and Danforth, 1997). Injection of recombinant bovine somatotropin into peripartum dairy cows improves the innate immune response and the IgG concentration of cows, but has limited influences on metabolism (Silva et al., 2015). In MDV infected cells, GH co-expresses with the proteins encoded by MDV serotype 1 (virulent) strains' SORF2 gene, and the polymorphism of GH gene is associated with the number of tissues with tumors in commercial White Leghorn chickens (Liu et al., 2001). In chicken bursa cells, the anti-apoptotic effect of GH is mediated by PI3K/Akt pathway (Luna-Acosta et al., 2015). There are evidences showing that GH is involved in the development, differentiation, and regulation of the immune response, and can be used as an immunomodulator.

Generally, GH exerts its function by regulating downstream gene expression in target cell that indirectly controls the expression of downstream genes. A previous study has shown that GH regulates cell growth, proliferation, and tissue regeneration by affecting the expression of a series of genes related to cells growth (Rotwein and Chia, 2010). The mRNA expression of 8 genes, including *gp130, STAT4*, and *MAPK p38*, is upregulated after GH injection in pituitary deficient rats (Thompson et al., 2000). GH may protect lymphocytes from glucocorticoid-induced apoptosis by inhibiting the activation of NFκB signaling pathway, or activating NFκB signaling pathway to participate in autoimmune diseases (Jeay et al., 2001, 2002). Like PRL, GH also activates ERK, MAPK and PI3K signaling pathways (Halevy et al., 1996; O'Connor, 2003; Reindl et al., 2011).

**Figure 2:**
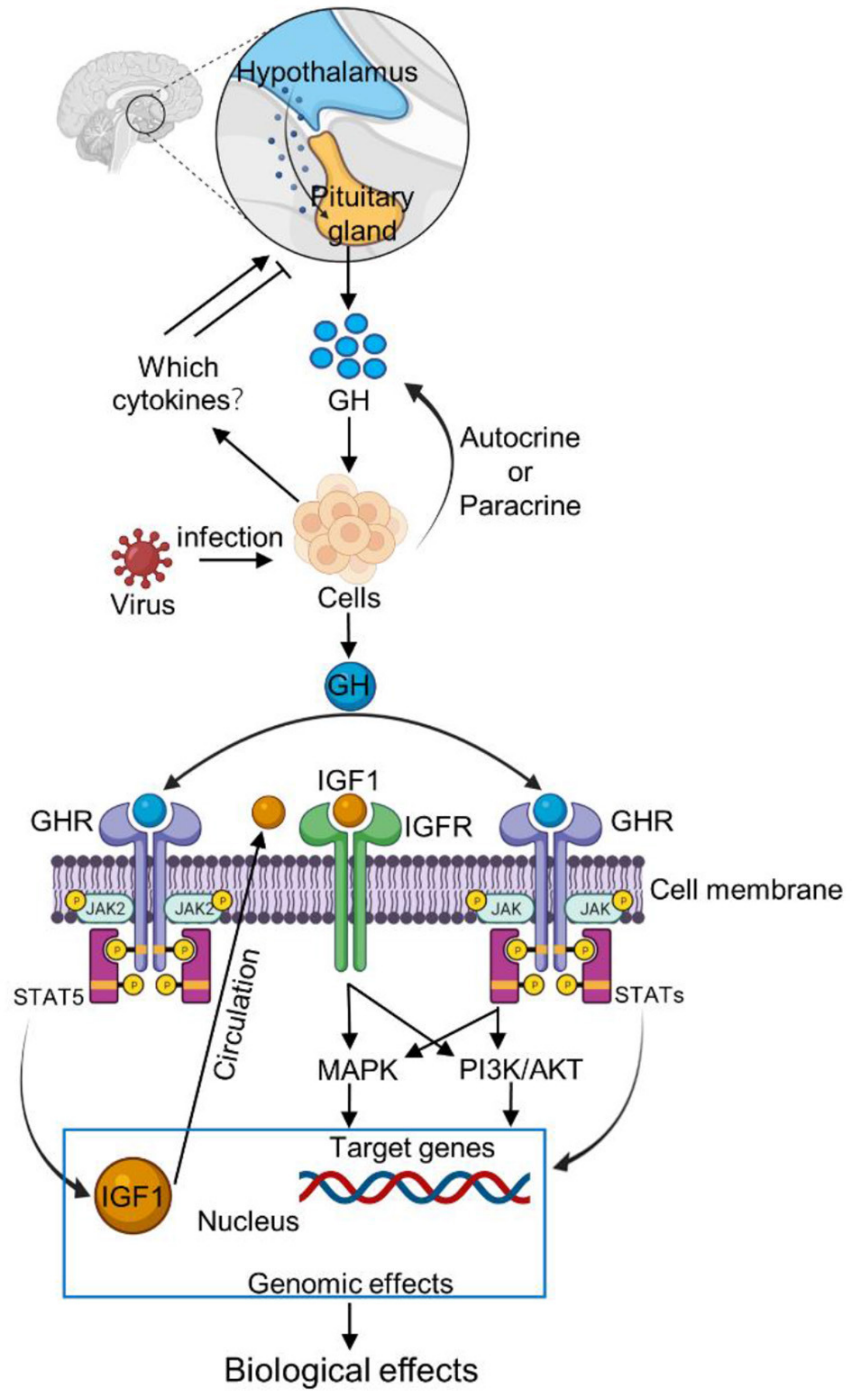
Schematic representation of GH signaling pathway after virus infection. The interactions between GH and cytokines are ambiguous and contradictory but the ghrelin can induce GH secretion. After virus infection, the host cells increase the expression of ghrelin. Ghrelin stimulates the pituitary gland to increase GH secretion. Meanwhile, cells can also increase GH secretion by autocrine or paracrine pathways. Then GH binds to the GHR receptor on the cell membrane, and activates JAK/STAT, MAPK and PI3K signaling pathways, thus regulating the individual immune response. Moreover, GH combined with GHR to activate the IGF1by inducing the JAK2/STAT5 in response to immune response.

GH has two distinct effects: a direct effect is GH binding to its receptor on target cells and an indirect effect is mediated primarily by insulin-like growth factor 1 (IGF1). The growth promoting effect of GH is mediated to a large extent by IGF1. GH/IGF1 has a prominent regulatory role in the immune response to infection, and mainly influences humoral and cellular functions (Heemskerk et al., 1999). IGF1 can regulate adaptive immunity, mainly by stimulating lymphopoiesis and increasing the responses to antigen-mediated activation. In mammals, IGF1 is an important cytokine that stimulates the host's immune response, including antibody production, lymphocyte proliferation, phagocytosis, and natural killer cell activity (Auernhammer and Strasburger, 1995; Jeay et al., 2002; Yada, 2007). GH combines with GHR to activate the synthesis of IGF1 by the phosphorylation of JAK2/STAT5 (Hu et al., 2021). It has been proven that GH, directly or indirectly through the IGF1, influences both cellular immunity and humoral immunity (Hakuno and Takahashi, 2018; Szalecki et al., 2018). These results suggest that some immune effects of GH may be regulated by IGF1. The GH-GHR-IGF1 axis has been recognized to play essential roles in somatic growth, including cell proliferation, differentiation, division, and survival. On the other hand, the GH-GHR-IGF1 axis also plays an important role in immune function, with regulatory mechanisms of unexpected complexity and versatility (Figure 2) (Hu et al., 2021).

Meanwhile, GH also affects the progress of autoimmune disease. GH slows the progression of diabetes by reducing apoptosis and/or increasing the proliferation rate of insulin-producing β cells (Villares et al., 2013). When individual against bacterial infection, GH can enhance the phagocytic activity, killing and elimination of bacteria *via* increasing opsonic activity (Saito et al., 1996). However, whether there is a similar response when the chickens encounter a virus infection remains unknown.

Immune cells can secrete numerous bioactive cytokines which affect neuroendocrine processes, but the activity of immune system is also modulated by cytokines (Pagani et al., 2005). The interactions between GH and cytokines are ambiguous and contradictory in the available literature (Szalecki et al., 2018). During viral infection, the mechanism of feedback to the hypothalamus remains unclear. Studies have shown that the expression of ghrelin in plasma and bursa of fabricius is upregulated in virus-infected individuals (Yu et al., 2019). Most importantly, ghrelin can induce GH secretion (Reichenbach et al., 2012). But not all virus increases the expression of ghrelin. We support the hypothesis that the combination of GH and GHR might activate JAK/STAT, MAPK or PI3K signaling pathways to regulate the host immune response after viral infection (Figure 2).

## PRLR and GHR as Potential Viral Receptors

Before invading host cells, viruses need to combine or adhere to receptors on the cell surface. There are many receptors on host cell membranes that can combine with viruses, including lipids, glycoproteins, growth factors, and hormone receptors (Grove and Marsh, 2011; Backovic and Rey, 2012; Wallis, 2021). These receptors have normal cellular functions on the surface of host cells, and can be hijacked by viruses to assist them in infecting cells (Kaelber et al., 2012; Demogines et al., 2013). In poultry, there are many cell surface receptors or other factors that can be utilized by viruses (Table 1).

**Table 1:** Poultry-related virus receptors.

**Virus**	**Receptor**	**References**
ALVs	chNHE1, Tva/Tvb/Tvc, chANXA2, chGRP78	Crittenden et al., 1967; Chai and Bates, 2006; Mei et al., 2015; Wang et al., 2016; Guan et al., 2017
IBV	Sialic acids, HSPA8	Winter et al., 2006; Zhu et al., 2020
NDV	Sialic acids	Helen et al., 2002; Guo et al., 2018
IBDV	c-Src, CD74	Ye et al., 2016; Liu et al., 2020

*ALV, Avian leucosis virus; IBV, Infectious bronchitis virus; NDV, Newcastle disease virus; IBDV, Infectious bursal disease virus; chNHE1, Chicken sodium hydrogen exchanger type 1; Tva/Tvb/Tvc, Tumor virus A/B/C; chANXA2, Chicken Annexin A2; chGRP78, Chicken glucose-regulation protein 78; HSPA8, Heat Shock Protein Member 8*.

In order to enter a target cell, the virus first attaches to a receptor on the cell's surface. The interaction between viruses and the host's receptor proteins may lead to an “arms race” of host-virus (Coffin, 2013; Wang et al., 2020). In this process, both host and virus may undergo mutations that inhibit or enhance their effects, then leading to rapid co-evolution of host's receptors and virus. The interactions between pathogens and hormones often play a significant role in “arms race” (Daugherty and Malik, 2012; Coffin, 2013; Wallis, 2021). PRLR and GHR show periodic evolutionary patterns, which are most obvious in the extracellular domain (Li et al., 2005). The rapid evolution of PRLR and GHR may reflect the host attempt to limit the use of PRLR and GHR as receptors by viruses to enter target cells (Wallis, 2021). Some viruses produce proteins, which are similarly to peptide hormones in structures, are beneficial for virus survival and growth in cells (Altindis et al., 2018; Huang et al., 2019). There is evidence that these proteins can be used by viruses as ligands for hormone receptors on host cells (Huang et al., 2019; Irwin, 2019). Thus, PRLR and GHR might provide a potential pathway for virus entry into host cells.

PRL and GH receptors are belonged to the cytokine/hematopoietic receptor superfamily. PRLR and GHR are expressed in a variety of immune cells, including monocytes, macrophages, lymphocytes, and natural killer cells (Borba et al., 2018; Wallis, 2021). Notably, PRLR and GHR are unable to initiate an immune response by themselves, and they need to combine with specific hormones to activate the relevant signaling pathways. They are closely related to JAK/STAT, MAPK, and PI3K/AKT signaling pathways (Bolefeysot et al., 1998).

There is evidence that the late-feathered (LF) chickens are more susceptible to ALV-J than the early-feathered (EF) chickens (Harris et al., 1984; Fadly and Smith, 1997). LF chickens have one more fusion gene, *dSPEF2/dPRLR*, and endogenous retroviruses 21 (*ev21*) compared with EF chickens (Elferink et al., 2008; Luo et al., 2012). *dSPEF2/dPRLR* is responsible for the expression of late feathering in chickens, but not the ev21 gene (Takenouchi et al., 2018). Furthermore, the *dPRLR* gene encodes a new PRL functional receptor that is widely expressed in all chicken tissues, and the pattern of spatiotemporal expression is likely to match that of the original *PRLR* gene (Bu et al., 2013). We speculate that LF chickens are susceptible to ALV-J because they carry both *PRLR* and *dPRLR* genes.

The combination of GH and GHR can activate the phosphorylation of JAK/STAT signaling pathway, especially STAT5. The phosphorylation of STAT5 activates the expression of IGFs which have been shown to play an important role in the immune system (Buuloffers and Kooijman, 1998; Jensen et al., 2005). However, some IGFs are receptors for virus entry into cells. For example, IGF1R has been shown to be a receptor for the respiratory syncytial virus (RSV) (Griffiths et al., 2020). Based on the above studies, it is speculated that PRLR and GHR may be the receptors for virus invasion of host cells.

## Conclusion and Perspective

Viral infection not only triggers an innate antiviral response, but also inhibits HPA activation in host (Pearce et al., 2001; Silverman et al., 2005). Inhibition of HPA activation results in increased immune/inflammatory reactivity and the severity of infection (Bailey et al., 2003; Webster and Sternberg, 2004). Host serum hormone levels will be influenced after infection with the pathogen, and then the various host physiological processes will also be affected (Rousseau, 2013; Mayer et al., 2019; Liu et al., 2021). In recent years, several studies have revealed the mechanisms on how innate antiviral response is regulated by hormones, such as melatonin, PRL, and progesterone (Hartwell et al., 2014; Morchang et al., 2021; Su et al., 2022). So far, how hormones regulate innate antiviral immunity remains unknown.

Individuals with high PRL levels have relatively strong resistance to pathogens (Mohammadpour et al., 2018; Mo et al., 2021). Increasing the plasma PRL levels of host can obviously improve its resistance to infection diseases (Mo et al., 2021). After virus infection, the changes of hormonal levels affect the disappearance of viremia in serum and the expression of antiviral genes in immune organs (Dai et al., 2019; Mo et al., 2021). In addition, PRL and GH also promote the proliferation and maturation of immune cells, and inhibit the expression of inflammatory factors (SkwarłoSońta, 1990; Marsh et al., 1992; Johnson et al., 1993; Mo et al., 2021). Interestingly, PRL expression is down-regulated, while GH expression is up-regulated after virus infection (Borghetti et al., 2011; Saleri et al., 2019; Mo et al., 2021, 2022). These results suggest that PRL and GH may play a compensatory role in the chicken immune system. Although their main functions are different, but they both regulate immunity through JAK/STAT signaling pathways.

For each pathogen, we always focused on the transmissibility, virulence and damage of virus, the immune response of the host, and the related prevention and control measures for the infectious diseases of poultry. PRL and GH are usually taken as the factors associated with the growth or breed of animals rather than important factors involved in the defense against the infections. However, current research results suggest that PRL and GH and their receptors are also an important factor in the individual defense system (SkwarłoSońta, 1990; Mo et al., 2021; Wallis, 2021). Although the mechanism of PRL and GH involvement in immunity is not fully understood, there is compelling evidence that they affect the communication and regulation of immune response (SkwarłoSońta, 1990; Skwaro-Sońta, 1992; Tang et al., 2017).

PRL and GH affect the immune system in many ways, which may be caused by the differences between cells, tissues, organs and species or the various physiological conditions *in vivo* and *in vitro* (Bhat et al., 1983; SkwarłoSońta, 1990; Allen et al., 1997; Filipin et al., 2019; Mo et al., 2021). Besides, we elucidate the potential effects of the receptors of PRL and GH on the immune system from another aspect, which makes us think deeply about the multiple roles that their receptors may play on the host immune system. It is also possible that the virus first directly binds to the hormone or adsorbed to the hormone surface, and then interacts with the hormone receptor, such as endocytosis. The virus takes the opportunity to enter the cell and start self-replication to injure the host.

PRL plays an important role in human autoimmune diseases (Vera-Lastra and Jara, 2002; Borba et al., 2018; Borba and Shoenfeld, 2019). However, its mechanism of action remains unclear, and even less understood in poultry. Much is known about the chemical, biological and reproductive regulation of PRL and GH in poultry (Wilkanowska et al., 2014), but few about the immune function *in vivo*. Here, we describe PRL and GH along with their receptors in terms of immunity, and reminding the scientists engage in poultry disease research that the hormones are an important factor in the immune response to against diseases.

Deciphering the multi-faceted influences of PRL, GH and their receptors on the responsiveness of immune system might be critical in elucidating key pathogenic mechanisms and modulates host innate antiviral response after virus infection. Overall, the intersection of avian immunology and endocrinology is a relatively new field of study, and there are still much more researches needs to be done.

## Author Contributions

GM designed the framework of the draft and wrote the manuscript. BH, PW, and QL helped by providing useful discussion and language correction. XZ revised and approved the final manuscript. All authors read and approved the final manuscript.

## Funding

This work was supported by the National Natural Science Foundation of China (31970540 and 31801030) and the China Agriculture Research System of MOF and MARA (CARS-41).

## Conflict of Interest

The authors declare that the research was conducted in the absence of any commercial or financial relationships that could be construed as a potential conflict of interest.

## Publisher's Note

All claims expressed in this article are solely those of the authors and do not necessarily represent those of their affiliated organizations, or those of the publisher, the editors and the reviewers. Any product that may be evaluated in this article, or claim that may be made by its manufacturer, is not guaranteed or endorsed by the publisher.
